# Relationship between family-related factors and functional constipation among Chinese preschoolers: a case–control study

**DOI:** 10.1186/s12887-022-03521-w

**Published:** 2022-08-01

**Authors:** Yushuo Niu, Ting Liu, Ni Ran, Kuinan Li, Yaru Sun, Xin Wang, Kun Guo, Xiuling Yang

**Affiliations:** 1 School of Nursing, Qingdao University, No.38 Dengzhou Road, Qingdao, 266021 Shandong Province China; 2 The Affiliated Hospital of Qingdao University, Qingdao, Shandong Province China

**Keywords:** Functional constipation, Preschoolers, Family, Risk factors

## Abstract

**Background:**

Constipation is one of the common symptoms in childhood. The prevalence of FC is about 0.5% to 32% and still on the rise according to global statistics. The aim of this study is to explore the associations between family-related factors (e.g., parental conflict, parenting style, and parent–child relationship) and functional constipation of preschool children based on family system theory.

**Methods:**

The study is a case–control survey of preschoolers in China. In total, 108 preschoolers with functional constipation diagnosed with pediatric Rome IV criteria and 324 healthy examination preschoolers without functional constipation were enrolled in the study. Parents completed the following 5 instruments: General information questionnaire, the Parental Conflict Scale, the Parenting Style Questionnaire, the Child-parent Relationship Scale and the Children’s Emotional Adjustment Scale-Preschool Version.

**Results:**

Nine categories of factors which significantly predicted functional constipation in preschoolers were retained in the final logistic regression model: Second child in birth order (OR = 0.456; 95% CI, 0.229 to 0.910), children picky eating (OR = 2.936; 95% CI, 1.133 to 7.611), bad bowel habits (OR = 2.896; 95% CI, 1.391 to 6.028), parental history of constipation (OR = 3.259; 95% CI, 1.600 to 6.639), parents blaming the child for having a bad bowel movement (OR = 3.788; 95% CI, 1.391 to 10.318), more than 3 h of fathers-child interaction time per day (OR = 0.137; 95% CI, 0.024 to 0.778), parental conflict (OR = 1.981; 95% CI, 0.950 to 3.831), doting or authoritarian parenting style (OR = 1.644; 95% CI, 1.067 to 2.534, OR = 2.481; 95% CI, 1.362 to 4.519), and anxiety control or temper control in children (OR = 0.492; 95% CI, 0.303 to 0.799, OR = 0.189; 95% CI, 0.103 to 0.348).

**Conclusions:**

This study identified the significant associations between family-related factors and functional constipation in preschool children, which provide implications for healthcare professionals to address functional constipation in early childhood using a preventive lens.

**Supplementary Information:**

The online version contains supplementary material available at 10.1186/s12887-022-03521-w.

## Background

Constipation is one of the common symptoms in childhood [1]. Over 95% of children with constipation have no organic cause, which is diagnosed as functional constipation (FC) according to the Rome IV criteria [2]. The prevalence of FC is about 0.5% to 32% and still on the rise according to global statistics [3]. Although FC rarely leads to life-threatening complications, it can cause physical pain and emotional distress in children [4]. Specifically, children with FC show more aggressive behaviors and other problems such as low self-esteem, anxiety, depression, poor attention and low social competency [5]. Furthermore, FC also causes concern for parents, ultimately impairing their health related quality of life (HRQOL) [6]. In addition, approximately 50% of children with FC were cured after 6–12 months of treatment, but 40% of patients still developed symptoms after treatment [7]. Long-term follow-up study showed that about one-third of children FC and related symptoms persist into adulthood [8].

Exploring the risk factors of FC may help to better identify the complex pathophysiology and optimize the intervention approaches. Previous research found that multiple factors (e.g., dietetic and bowl evacuation habit, and psychosocial issues) played a role in the persistence of symptoms of the constipated children [9–11]. Among these factors, family-related psychosocial issues such as parental factors, early life-events, and the child's psychological state were considered as contributing factors of pathophysiology of FC [8]. Preschool age is an important period for children development, and the family is the primary environment for preschoolers’ socialization [12]. Family system theory stated that family is composed of various subsystems (e.g., the conjugal subsystem, the parent subsystem, and the child-parent subsystem), and the interaction among the subsystems affects the development of the individual [13]. Therefore, identification of significant family-related factors could shed light on the etiology of childhood FC and thus inform related prevention strategies. However, family-related factors were often measured by a single item (e.g., frequency of interaction with parents, or punishment) and few studies focused on the effect of the interaction between family members on FC in China, and thus could not capture a comprehensive picture of the relationship between childhood FC and family system.

Based on family system theory, this study aims to explore the associations between family-related factors (e.g., parental conflict, parenting style, and parent–child relationship) and FC of preschool children by using a case–control study design, with a focus on assessment of the association strength.

## Methods

### Sample size calculation

The formula was used to calculate sample size: $\mathrm{n}=\frac{(1+1/\mathrm{c})\mathrm{\overline{p}}\overline{q}{({\mathrm{Z}}_{\mathrm{\alpha}}{\mathrm{Z}}_{\upbeta})}^{2}}{{({\mathrm{p}}_{1-}{\mathrm{p}}_{0})}^{2}}$, where P_1 =_ $\frac{{ORP}_{0}}{1+{P}_{0}(OR-1)}$, $\overline{p}=\frac{{\mathrm{p}}_{1}+{\mathrm{cp}}_{0}}{1+\mathrm{c}}$, $\overline{q}= 1- \overline{p}, {\mathrm{Z}}_{\mathrm{\alpha }}$ = 1.96, ${\mathrm{Z}}_{\upbeta } = 1.282$. According to the previous study (P_0_ = 0.2365, OR = 2) [14], the sample size of case group was 98. Moreover, a rough estimation by multiplying the sample size by 1.1 to 1.2 times to reduce the sampling error.

### Participant recruitment

The study was approved by the medical ethics review board (QYFY WZLL 25,896). The study was conducted in the Children Preventive Health Care Clinic of a tertiary hospital in an east coastal city of Shandong Province, China. Children and their parents, who were seeking primary care services at the Children Preventive Health Care Clinic from August 2020 to May 2021, were invited to participate in the study. Written informed consent for participation was obtained from parents. Inclusion criteria were as follows: (1) children diagnosed as FC by pediatric Rome IV criteria; (2) children aged between 2 and 6 years; (3) parents aged ≥ 18 years; and (4) parents with literate and able to complete the questionnaire themselves. Exclusion criteria were as follows: children (1) with other diseases that cause constipation, such as congenital megacolon; (2) with other serious diseases, such as hematological and neoplastic disorders; and (3) participated in other clinical trials. There were 116 children and their parents who were eligible for enrollment in the study, and eight children were excluded because children’s parents did not complete the questionnaire. Finally, data from a total of 108 children were analyzed. Taking children's age as the matching factor, 324 parents of healthy examination children without FC were selected in the same clinic and the same period at a ratio of 1:3 as the control group based on the reported FC incidence data in China [14].

Questionnaires were collected by trained investigators in the form of face-to-face interviews. Parents were asked to complete the questionnaire for their children. Prior to the survey, the investigators explained the purpose of the study to the participants. Each survey took about 20 min to complete. To avoid missing data, the investigators checked the questionnaires one by one and asked the parents to fill in the missing items with their willingness.

### Measures

Children’s parents completed the following 5 questionnaires: General information questionnaire, the Parental Conflict Scale, the Parenting Style Questionnaire, the Child-parent Relationship Scale and the Children's Emotional Adjustment Scale-Preschool Version.

### General information questionnaire

Based on literature review and consulting experts in the field of child health care and gastroenterology, a general information questionnaire was designed. The questionnaire included three parts: (1) demographic information about age, gender, birth order, family type, mother's working status, monthly household income and parent's highest level of education; (2) children's factors including food allergy, poor appetite, and bowel habits, etc.; and (3) family related factors including parents' history of constipation, interaction time between parents and children, etc.

### The parental conflict scale

The Parental Conflict Scale (PCS) was used to evaluate the intensity of parental conflict, which was designed by Chen et al. [15] in 2015. It includes 5 items, which are self-evaluated by parents and has been widely used as a validated tool in various studies with good reliability and validity. Items are scored on a five-point Likert scale, which ranges from 0 (completely disagree), not at all to 5 (completely agree), with higher scores indicating higher parents conflict. The internal consistency was good in this study (Cronbach’s alpha 0.859).

### The parenting style questionnaire

The parenting style was measured using the Chinese version of the parental style questionnaire, which was designed by Yang [16].This instrument includes 40 items in five subscales: doting, permissive, authoritarian, democratic and inconsistent parenting style. Items are scored on a five-point Likert scale, ranging from 1 (never) to 5 (always). The higher score of the subscale indicates more popularity of parenting style in the subscale. The questionnaire demonstrated an acceptable internal consistency with Cronbach's alpha value of 0.879 and the subscales varied from 0.808 to 0.944 in the current study.

### The child-parent relationship scale

Child-parent relationship was assessed through the Child-parent Relationship Scale (CRS), which was translated and revised by Zhang et al. [17]. CRS is a 26-item scale that includes three dimensions: intimacy, dependence and conflict. Zhang et al. [17] reported that the reliability of dependency dimension was low, thus only two dimensions of intimacy and conflict were used in the present study. Items are scored on a 5-point Likert scale, with higher scores indicating higher intimacy or conflict in the child-parent relationship. The Cronbach's alpha was 0.812 for the total scale and was 0.802 and 0.905 for the two dimensions, respectively.

### The children’s emotional adjustment scale

The Children's Emotional Adjustment Scale-Preschool version (CEAS-P) was used to measure the ability to regulate key emotions such as anger, fear and shyness of children. The scale was revised by Wu et al. [18] in 2020. CEAS-P includes 29-item on three dimensions of temper control, social assertiveness and anxiety control. The items are scored on a 5-point Likert scale, with 1–5 points from “never” to “always”. The Cronbach’s alpha was 0.937 for the total scale and was 0.906–0.925 for the three dimensions.

### Statistical analysis

All statistical analyses were performed using Statistical Package for Social Sciences (SPSS) version 21.0. Measurement data with non-normal distribution were presented as medians and quartiles, and the Mann–Whitney U test was used to analyze. Categorical variables were presented as frequency and percentage, and Chi-squared test or Fisher exact tests were applied for data comparison. Logistic regression analysis was used to determine independent risk factors for FC in preschoolers based on the Wald statistics using backward stepwise progression. All risk factors with *P* < 0.05 were included in the multivariate predictive model. Variance Inflation Factor (VIF) was used to test multicollinearity of variables in the model. GraphPad Prism software 9.0.2 was used to plot the regression forest map.

## Results

### Participant characteristics

A total of 108 patients with FC and 324 preschoolers in the control group were enrolled in the study. Demographic characteristics of enrolled children and parents were shown in Table 1. There was significant difference regarding the birth order of the child, but there was no difference in comparison of other demographic characteristics between the two groups. Other information of the preschoolers and parents was compared, and the results showed that food allergy, poor appetite, picky eating, eating habits, water quantity, daily screen time, bowel habits, toilet training, parents blaming the child for having a bad bowel movement, parental history of FC, father-child interaction time and family relationships were associated with FC (*P* < 0.05) (Table 1).

**Table 1:** Characteristics of the study sample

Characteristics		FC group (*n* = 108)	Control group (*n* = 324)	*χ*^*2*^	*P* value
**Demographic information**					
Children’s age	2 years old	8 (7.4%)	27 (8.3%)	1.299	0.254
	3 years old	14 (13.0%)	53 (16.4%)		
	4 years old	34 (31.5%)	104 (32.1%)		
	5 years old	20 (18.5%)	62 (19.1%)		
	6 years old	32 (29.6%)	78 (24.1%)		
Children’s gender	Male	54 (50.0%)	172 (53.1%)	0.309	0.578
	Famale	54 (50.0%)	152 (46.9%)		
Children’s birth order	First child	69 (63.9%)	157 (48.5%)	7.746	0.021
	Second child	35 (32.4%)	151 (46.6%)		
	Third child and above	4 (3.7%)	16 (4.9%)		
Father’s education level	Junior high or below	27 (25.0%)	108 (33.3%)	5.125	0.163
	High school/technical secondary school	31 (28.7%)	65 (20.1%)		
	College degree	24 (22.2%)	63 (19.4%)		
	Bachelor degree or above	26 (24.1%)	88 (27.2%)		
Mother’s education level	Junior high or below	29 (26.9%)	107 (33.3%)	1.638	0.651
	High school/technical secondary school	28 (25.9%)	71 (21.9%)		
	College degree	21 (19.4%)	61 (18.8%)		
	Bachelor degree or above	30 (27.8%)	85 (26.2%)		
Mother’s working status	Unemployed	31 (28.7%)	113 (34.9%)	2.513	0.285
	A part-time job	8 (7.4%)	32 (9.9%)		
	A full-time job	69 (63.9%)	179 (55.2%)		
Family structure	Big family	19 (17.6%)	75 (23.1%)	5.177	0.270
	The expanded nuclear family	33 (30.6%)	80 (24.7%)		
	The nuclear family	53 (49.1%)	166 (51.2%)		
	Reorganization of the family	1 (0.9%)	2 (0.6%)		
	Incomplete family	2 (1.9%)	1 (0.3%)		
The average monthly income of a family	< 3000 yuan	3 (2.8%)	6 (1.9%)	4.319	0.229
	3000–5000 yuan	37 (34.3%)	84 (25.9%)		
	5001–10,000 yuan	52 (48.1%)	192 (59.3%)		
	> 10,000 yuan	16 (14.8%)	42 (13.0%)		
**Possible risk factors associated with functional constipation**					
Parental history of constipation	Yes	44 (540.7%)	275 (84.9%)	31.466	< 0.001
	No	64 (59.3%)	49 (15.1%)		
Food allergies	Yes	18 (16.7%)	28 (8.6%)	5.482	0.019
	No	90 (83.3%)	296(91.4%)		
Inappetence	Yes	22 (20.4%)	21 (6.5%)	17.433	< 0.001
	No	86 (79.6%)	303 (93.5%)		
Bad eating habit	Yes	51 (47.2%)	53 (16.4%)	42.214	< 0.001
	No	57 (52.8%)	271 (83.6%)		
Picky and partial eaters	Yes	76 (70.4%)	15 (4.6%)	52.212	< 0.001
	No	32 (29.6%)	309 (95.4%)		
Water quantity	< 400 ml	24 (22.2%)	65 (20.1%)	13.358	0.004
	400-600 ml	60 (55.6%)	174 (53.7%)		
	601-800 ml	17 (15.7%)	40 (12.3%)		
	> 800 ml	7 (6.5%)	45 (13.9%)		
Daily activity time (outdoor + indoor)	≦1 h	13 (12.0%)	46 (14.2%)	0.389	0.823
	2-4 h	38 (35.2%)	107 (33.0%)		
	≧5 h	57 (52.8%)	171 (52.8%)		
Daily screen time	< 30 min	43 (39.8%)	169 (52.2%)	11.116	0.004
	30–60 min	34 (31.5%)	107 (33.0%)		
	> 60 min	31 (28.7%)	48 (14.8%)		
Bad bowel habits	Yes	47 (43.5%)	44 (13.6%)	43.663	< 0.001
	No	61 (56.5%)	280 (86.4%)		
Time to toilet training	No bowel movement training	55 (50.9%)	151 (46.6%)	0.667	0.881
	< 1 year old	18 (16.7%)	56 (17.3%)		
	1–2 years old	27 (25.0%)	89 (27.5%)		
	> 2 years old	8 (7.4%)	28 (8.6%)		
Blaming the child for having a bad bowel movement	Yes	31 (28.7%)	12 (3.7%)	56.483	< 0.001
	No	77 (71.3%)	312 (96.3%)		
Daily father-child interaction time	< 1 h	37 (34.3%)	183 (56.5%)	12.853	0.002
	1-3 h	69 (63.9%)	106 (32.7%)		
	> 3 h	2 (1.9%)	35 (10.8%)		
Daily mother–child interaction time	< 1 h	13 (12.0%)	58 (17.9%)	12.259	0.002
	1-3 h	52 (48.1%)	123 (38.0%)		
	> 3 h	43 (39.8%)	143 (44.1%)		

*FC* Functional constipation

### Parental conflict

The total score of parental conflict were significantly different between the two groups (*P* < 0.05) (Table 2). Parents of children with FC reported significantly more conflict compared with parents of controls (*P* < 0.05).

**Table 2:** Comparison of mean scores of parental conflict, parenting style, child-parent relationship and children’s emotional adjustment between two groups

Instrument		FC group (*n* = 108)	Control group (*n* = 324)	*P* value*
Parental conflict		2.40 (2.00, 2.80)	1.80 (1.00, 2.20)	< 0.001
Parenting style	Doting	1.64 (1.14, 2.42)	1.21 (1.00, 1.86)	< 0.001
	Democratic	3.90 (3.70, 4.00)	4.00 (3.80, 4.20)	< 0.001
	Permissive	1.72 (1.33, 2.11)	1.56 (1.11, 2.00)	0.010
	Authoritarian	2.75 (2.41, 3.25)	2.50 (2.00, 3.00)	< 0.001
	Inconsistent	2.67 (1.88, 3.00)	2.17 (1.50, 3.00)	0.002
Child-parent relationship	Intimacy	4.40 (4.00, 4.68)	4.50 (4.20, 4.80)	0.004
	Conflict	3.17 (2.75, 3.81)	2.38 (1.92, 3.00)	< 0.001
Children’s emotional adjustment	Social assertiveness	2.85 (2.40, 3.20)	3.40 (2.93, 3.90)	< 0.001
	Temper control	2.71 (2.29, 3.00)	3.43 (3.00, 4.00)	< 0.001
	Anxiety control	2.55 (1.93, 3.00)	3.18 (2.91, 3.73)	< 0.001

*FC* Functional constipation

^*^P values were derived from Mann–Whitney U tests on data

### Parenting style

There were significant differences in scores between the two groups on all five parenting style dimensions (*P* < 0.05) (Table 2). Specifically, the scores of parents in the FC group were higher than those in the control group in doting, permissive, authoritarian and inconsistent parenting style, while lower in the democratic parenting style.

### Child-parent relationship

The scores of child-parent relationships were significantly different between the two groups (*P* < 0.05) (Table 2). The scores of conflict between parents and FC children was significantly higher than that in the control group, while the intimacy was opposite.

### Children’s emotional adjustment

There were significant differences in scores between the two groups on all three dimensions of temper control, social assertiveness and anxiety control (*P* < 0.05) (Table 2). The ability of social assertiveness, temper control and anxiety control in the FC group was significantly lower than that in the control group.

### Predictive variables of children FC

**Fig. 1:**
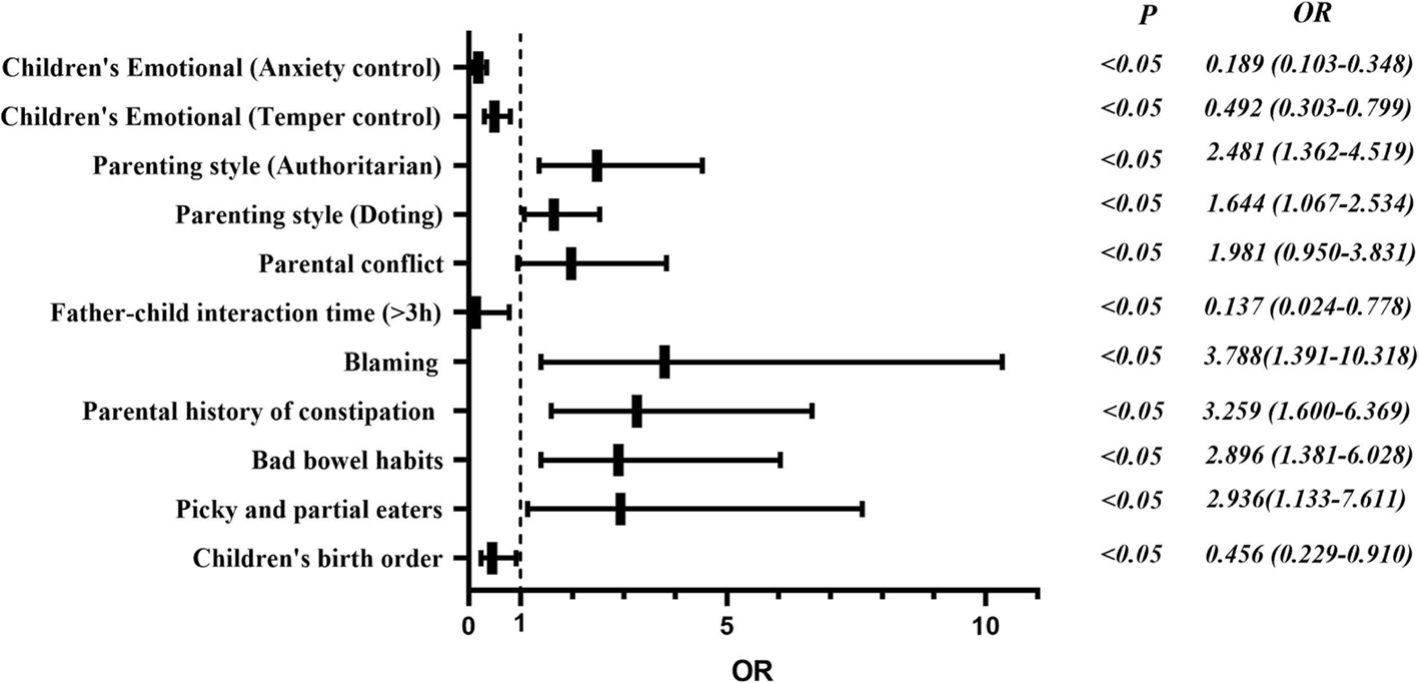
The forest map to predict functional constipation in children

With independent variables confirmed as being statistically associated with preschoolers FC in preschoolers, multiplicity tests should be done for the significance. The results show that there is no multicollinearity between variables (VIF < 10) (Table 3). Then, multivariate analysis was conducted. Nine categories of risk factors which significantly predicted FC in preschoolers were retained in the final logistic regression model (Table 4, Fig. 1).

**Table 3:** Test for multicollinearity between variables

Characteristics		VIF
Children's birth order	First child	1.119
	Second child	
	Third child and above	
	A part-time job	
	A full-time job	
Parental history of constipation	Yes	1.095
	No	
Food allergies	Yes	1.152
	No	
Inappetence	Yes	1.320
	No	
Bad eating habit	Yes	1.434
	No	
Picky and partial eaters	Yes	1.413
	No	
Water quantity	< 400 ml	1.112
	400-600 ml	
	601-800 ml	
	> 800 ml	
Daily screen time	< 30 min	1.201
	30–60 min	
	> 60 min	
Bad bowel habits	Yes	1.219
	No	
Blaming the child for having a bad bowel movement	Yes	1.295
	No	
Daily father-child interaction time	< 1 h	1.210
	1-3 h	
	> 3 h	
Daily mother–child interaction time	< 1 h	1.249
	1-3 h	
	> 3 h	
Parental conflict		1.720
Parenting style	Doting	1.455
	Democratic	1.357
	Permissive	1.578
	Authoritarian	1.510
	Inconsistent	1.418
Child-parent relationship	Intimacy	1.363
	Conflict	1.859
Children’s emotional adjustment	Social assertiveness	1.642
	Temper control	1.864
	Anxiety control	1.663

**Table 4:** Logistic regression models predicting Children functional constipation

Predictive factor	B	SE	Wald	*P*	OR (95% CI)
Children’s birth order (First child)			5.597	0.061	
Second child	-0.785	0.352	4.963	0.026	0.456 (0.229–0.910)
Third child and above	-0.874	0.684	1.632	0.201	0.417 (0.109–1.595)
Picky and partial eaters (No)					
Yes	1.077	0.486	4.914	0.027	2.936 (1.133–7.611)
Bad bowel habits (No)					
Yes	1.063	0.374	8.084	0.004	2.896 (1.391–6.028)
Parental history of constipation (No)					
Yes	1.181	0.363	10.587	0.001	3.259 (1.600–6.639)
Parents blaming the child for having a bad bowel movement (No)					
Yes	1.332	0.511	6.788	0.009	3.788 (1.391–10.318)
Daily father-child interaction time (< 1 h)			5.052	0.080	
1-3 h	-0.148	0.365	0.164	0.685	0.862 (0.422–1.763)
> 3 h	-1.987	0.886	5.032	0.025	0.137 (0.024–0.778)
Parental conflict	0.684	0.247	7.663	0.006	1.981 (0.950–3.831)
Parenting style (Doting)	0.497	0.221	5.071	0.024	1.644 (1.067–2.534)
Parenting style (Authoritarian)	0.909	0.306	8.812	0.003	2.481 (1.362–4.519)
Children’s Emotional (Temper control)	-0.709	0.247	8.222	0.004	0.492 (0.303–0.799)
Children’s Emotional (Anxiety control)	-1.665	0.312	28.488	< 0.001	0.189 (0.103–0.348)
Constant	-2.140	1.902	1.266	0.260	0.118

N = 432 children ages 2–6 years

## Discussion

This study focused on the effects of family-related factors on FC in preschoolers. As hypothesized, significant associations between parental conflict, parenting style, children’s emotional adjustment and FC in preschoolers were identified. Unexpectedly, there was no significant link between child-parent relationship and FC in preschoolers.

Children with FC were reported to score higher on parental conflict in the current study. Devanarayana et al. [19] found that domestic violence was associated with higher rates of constipation in children, which supports the current results. Children are highly sensitive and tend to internalize family stress and strain caused by parental conflict into their own emotions and behaviors, which increases the risk of poor physical and mental development [20]. Therefore, reducing the frequency and intensity of parental conflict could be served as a practical means of preventing FC in preschoolers. For this sense, healthcare professionals need to improve awareness of the potential impact of parental factors on constipation in children and advise parents to consciously pay attention to appropriately managing the conflicts between husband and wife, and creating a harmonious family atmosphere.

Parenting style is associated with constipation in children [21, 22]. The low autonomy scores in children were associated with decreased defecation frequency [22]. Likewise, authoritarian parenting was significantly associated with FC in children in the current study. Authoritarian parents are inclined to favor the compliance of children and over-restrain the autonomy of children [23]. However, self-determination theory emphasizes that by supporting satisfaction of individuals’ psychological need of autonomy, social environment can enhance human’s internal motivation, promote the internalization of external motivation, and ensure the healthy growth of human beings [24]. When the social environment cannot support the need of autonomy, children will experience excessive psychological burden, and thus have an increasing risk of FC [25]. Notably, doting parenting, which is characterized by over-protection or solicitousness, was also significantly linked to FC in the current study. The result adds a viewpoint to previous studies. This may be attributed to the fact that overindulged children are more likely to develop bad habits [26]. Furthermore, a spoiled preschooler appears to be more dependent on their parents. For example, when the environment changes from family to kindergarten, it is easy to cause children’s maladjustment to the environment, low self-efficacy, anxiety and other emotional and behavioral problems, which increases the risk of children FC [27]. In addition, democratic parenting style was not included in the final regression model in the study, but univariate analysis results showed that only democratic parenting style was associated with reduced FC risk. The democratic style is characterized by respect for children, autonomy, and the promotion of desirable behavior, and creating a democratic environment and emotional warmth [28]. The children in these households tend to develop good social skills and a healthy mental environment (e.g., good self-control, self-esteem and initiative), which reduces the risk of children FC [8, 29]. Therefore, education for parents to realize the importance of democratic parenting style may be an effective way to reduce the occurrence of FC in early childhood.

It is known that the child-parent relationship is the cornerstone of early childhood development and a good child-parent relationship has a positive influence on children’s physical and psychological health [30, 31]. Several previous studies have confirmed an association between child-parent relationship and FC in children. Specifically, children who had infrequent interaction with their parents and rarely have dinner with their parents were more likely to have FC [32, 33]. In contrast to other studies, no dimension of the child-parent relationship scale (CRS) was retained in the final logistic regression model in the current study. The differences between the results may be attributed to the fact that the CRS questionnaire is more comprehensive in screening for child-parent relationships compared with the single item used in previous studies. A larger sample size is therefore needed to verify the results in the future. In addition, it is noteworthy that more than 3 h of fathers-child interaction time each day was a protective factor for the occurrence of FC. To our knowledge, few previous studies have explored the role of fathers in children’s FC. However, father-child interaction makes a unique contribution to a child’s development and emotional well-being [34, 35]. For this reason, future study is needed to explore the role of fathers in FC in children.

It is noteworthy that children’s emotional adjustment was linked with FC. Our study reported that children with FC had lower temper and anxiety control than healthy children, in line with the findings of a longitudinal study that showed children who often lose their temper were more than twice as likely to have FC as normal children [36]. Moreover, the risk of FC was 3.788 times higher for children who were blamed for poor bowel movements than for those who were not blamed in the present study. Children who are criticized for defecating have negative emotions such as psychological stress and fear of defecating. Negative emotions regulate colon and rectum function through efferent pathways, thus leading to gastrointestinal dysfunction [8]. In turn, gastrointestinal dysfunction also brings emotional pain to children [4].Therefore, there may be a bidirectional link between children’s emotions and constipation. Accordingly, healthcare professionals can improve children's mental health to prevent and reduce the occurrence of FC.

In addition, the present study found that some non-psychological factors were closely related to FC, such as child’s birth order, family history, and diet and bowel habits. One unexpected finding was that the second child was a protective factor for FC. There are two possible reasons for the results. First, parents have some parenting experience when raising their second child, which can better promote children to develop good living habits. Second, previous studies showed that young children have less emotional and behavioral problems than older children, while children’s psychological status is closely related to the occurrence of FC [37]. Currently, China’s one-child policy has been replaced by a universal three-child policy, and the number of children in a family will continue to grow. Therefore, parents need to realize the impact of birth order on children’s FC. In addition, findings on the relationship between family history and constipation have been inconsistent worldwide [8]. Our results showed a familial clustering of FC. Specifically, children with parental history of FC were 3.259 times, more likely to develop FC than children without a family history, which was similar to the results of previous research [38]. However, there is no conclusive evidence that specific genes are involved in FC [8]. It means that the role of genetic factors in the pathophysiology of FC remains to be explored.

## Conclusions

In summary, the study results shed light on the association between parental conflict, parenting style, parent–child relationship and FC in preschool children, which provide implications for healthcare professionals to address FC in early childhood using a preventive lens. Specifically, a family-centered approach that encourages a two-generation strategy to underscore parental relationship, parent–child relationship, parenting style, children's emotional adjustment and living habits may be useful to reduce the risk of FC in preschool children. However, there are some limitations in the study. First, the caregivers included in the study were mainly mothers, and the sample size of fathers was too small to make meaningful comparisons. In the future, the sample size of fathers needs to be increased to explore the difference between fathers and mothers regarding children’s FC. Second, data reported by parents may differ from those reported by children, which may provide more socially desirable answers in the survey. Finally, this study could not state the causal association between family-related factors and FC due to the retrospective design. In the future, longitudinal prospective studies are required to answer this question on causality.

## Supplementary Information

**Additional file 1.** General information questionnaire.

**Additional file 2.** Test for normality of continuous variables.

## Data Availability

The datasets used and/or analyzed during the current study are available from the corresponding author on reasonable request.

## Abbreviation

FC: Functional constipation
