# Supplementary material for: Relationship between family-related factors and functional constipation among Chinese preschoolers: a case–control study
Source: BMC Pediatr. 2022 Aug 1;22:460. doi: 10.1186/s12887-022-03521-w (PMC9341050; doi:10.1186/s12887-022-03521-w)
Supplement: Supplementary file 1 — Additional file 1. General information questionnaire. [file 12887_2022_3521_MOESM1_ESM.docx]

**General Information Questionnaire**

**Demographic information**

1. Are you the child's father or mother

□ father □ mother

1. Children's age ＿＿＿＿＿
2. Children's gender

□ Male □ Famale

1. Children's birth order

□ First child □ Second child □ Third child and above

1. Father's education level

□ Junior high or below □ High school/technical secondary school

□ College degree □ Bachelor degree or above

1. Mother's education level

□ Junior high or below □ High school/technical secondary school

□ College degree □ Bachelor degree or above

1. Mother's working status

□ Unemployed □ A part-time job □ A full-time job

1. Your family structure

□ Big family □ The expanded nuclear family □ The nuclear family

□ Reorganization of the family □ Incomplete family

1. The average monthly income of a family (yuan)

□ <3000 □ 3000-5000 □ 5001-10000 □ >10000

**Possible risk factors associated with functional constipation**

1. One of the parents has a history of constipation

□ Yes □ No

1. Is your child allergic to certain foods

□ Yes＿＿＿＿＿ □ No

1. Does your child often have poor appetite

□ Yes □ No

1. Does your child have bad eating habits

□ Yes □ No

1. Does your child have the habit of picky and partial food

□ Yes □ No

1. Child's daily water intake

□ <400ml □ 400-600ml ☑ 601-800ml □ >800ml

1. Child's daily activity time (outdoor + indoor)

□ ≦1h □ 2-4h □ ≧5h

1. Child's daily screen time

□ <30 min □ 30-60 min □ > 60 min

1. Does your child have bad bowel habits

□ Yes □ No

1. When did you train your child to defecate

□ No bowel movement training □ < 1 year old □ 1-2 years old □>2 years old

1. Do you often blame children when their defecation is not going well

□ Yes □ No

1. Daily father-child interaction time

□ <1h □ 1-3h □ >3h

1. Daily mother-child interaction time

□ <1h □ 1-3h □ >3h
