# Supplementary material for: Relationship between family-related factors and functional constipation among Chinese preschoolers: a case–control study
Source: BMC Pediatr. 2022 Aug 1;22:460. doi: 10.1186/s12887-022-03521-w (PMC9341050; doi:10.1186/s12887-022-03521-w)
Supplement: Supplementary file 2 — Additional file 2. Test for normality of continuous variables. [file 12887_2022_3521_MOESM2_ESM.docx]

**Test for normality of continuous variables**

| **Instrument** | | ***P* value^1)^** | ***P* value^2)^** |
| --- | --- | --- | --- |
| Parental conflict |  | <0.001 | <0.001 |
| Parenting style | Doting | <0.001 | <0.001 |
|  | Democratic | <0.001 | <0.001 |
|  | Permissive | 0.001 | <0.001 |
|  | Authoritarian | <0.001 | <0.001 |
|  | Inconsistent | 0.003 | <0.001 |
| Child-parent relationship | Intimacy | <0.001 | <0.001 |
|  | Conflict | 0.200 | <0.001 |
| Children's emotional adjustment | Social assertiveness | 0.062 | <0.001 |
|  | Temper control | 0.055 | <0.001 |
|  | Anxiety control | 0.001 | <0.001 |

^1)^Normality evaluated for functional constipation group; ^2)^Normality evaluated for control group.
